# Platinum-Based Neoadjuvant Chemotherapy Before Radical Prostatectomy for Locally Advanced Prostate Cancer With Homologous Recombination Deficiency: A Case Report

**DOI:** 10.3389/fonc.2021.777318

**Published:** 2022-01-05

**Authors:** Junlong Zhuang, Shun Zhang, Xuefeng Qiu, Yao Fu, Shuyue Ai, Tingting Zhao, Yining Yang, Hongqian Guo

**Affiliations:** ^1^Department of Urology, Affiliated Drum Tower Hospital, Medical School of Nanjing University, Nanjing, China; ^2^Institute of Urology, Nanjing University, Nanjing, China; ^3^Department of Pathology, Affiliated Drum Tower Hospital, Medical School of Nanjing University, Nanjing, China; ^4^Department of Nuclear Medicine, Nanjing First Hospital, Nanjing Medical University, Nanjing, China; ^5^GloriousMed Clinical Laboratory (Shanghai) Co., Ltd., Shanghai, China

**Keywords:** prostate cancer, neoadjuvant chemotherapy, platinum, next-generation sequencing, homologous recombination defect

## Abstract

More emerging evidence showed that homologous recombination (HR) defect (HRD) may predict sensitivity to platinum agents in metastatic prostate cancer (PCa). Platinum-based neoadjuvant chemotherapy for PCa with HRD has not been reported. Here, we reported a man diagnosed as locally advanced PCa with high Gleason Score (5 + 5) and low PSA level (5.2 ng/ml). Next-generation sequencing (NGS) demonstrated HRD. He received six cycles of platinum-based neoadjuvant chemotherapy before radical prostatectomy (RP). Fifteen months after RP, his PSA level was still undetectable, and no imaging progression was found, indicating a potential role for platinum-based neoadjuvant chemotherapy in locally advanced PCa with HRD.

## Introduction

The incidence of prostate cancer (PCa) has been increasing and shows younger trend over the years in China, especially in the urban areas, possibly because of more active prostate-specific antigen (PSA) screening, improved biopsy techniques, or popular Westernized lifestyle (1). Most Chinese patients are diagnosed with high-risk disease, and they have higher rate of relapse and death after radical prostatectomy (RP) (2). Neoadjuvant therapy performed before RP intends to improve the surgery efficacy by reducing the tumor burden and minimal residual disease. However, the effectiveness of neoadjuvant therapy on PCa remains controversial.

Previous studies have identified that about 20%–30% of patients with primary PCa harbor alterations in genes involved in homologous recombination (HR) pathway, including BRCA2 (3, 4). Deleterious alterations of genes in HR are associated with aggressive disease and poor clinical outcomes (5, 6). More emerging studies also suggested that HR defect (HRD) may predict sensitivity to platinum agents or poly(ADP-ribose) polymerase (PARP) inhibitors in metastatic PCa (7–12). Here, we reported a patient with locally advanced PCa with HRD who responded outstandingly to platinum-based neoadjuvant chemotherapy.

## Case Presentation

**Figure 1:**
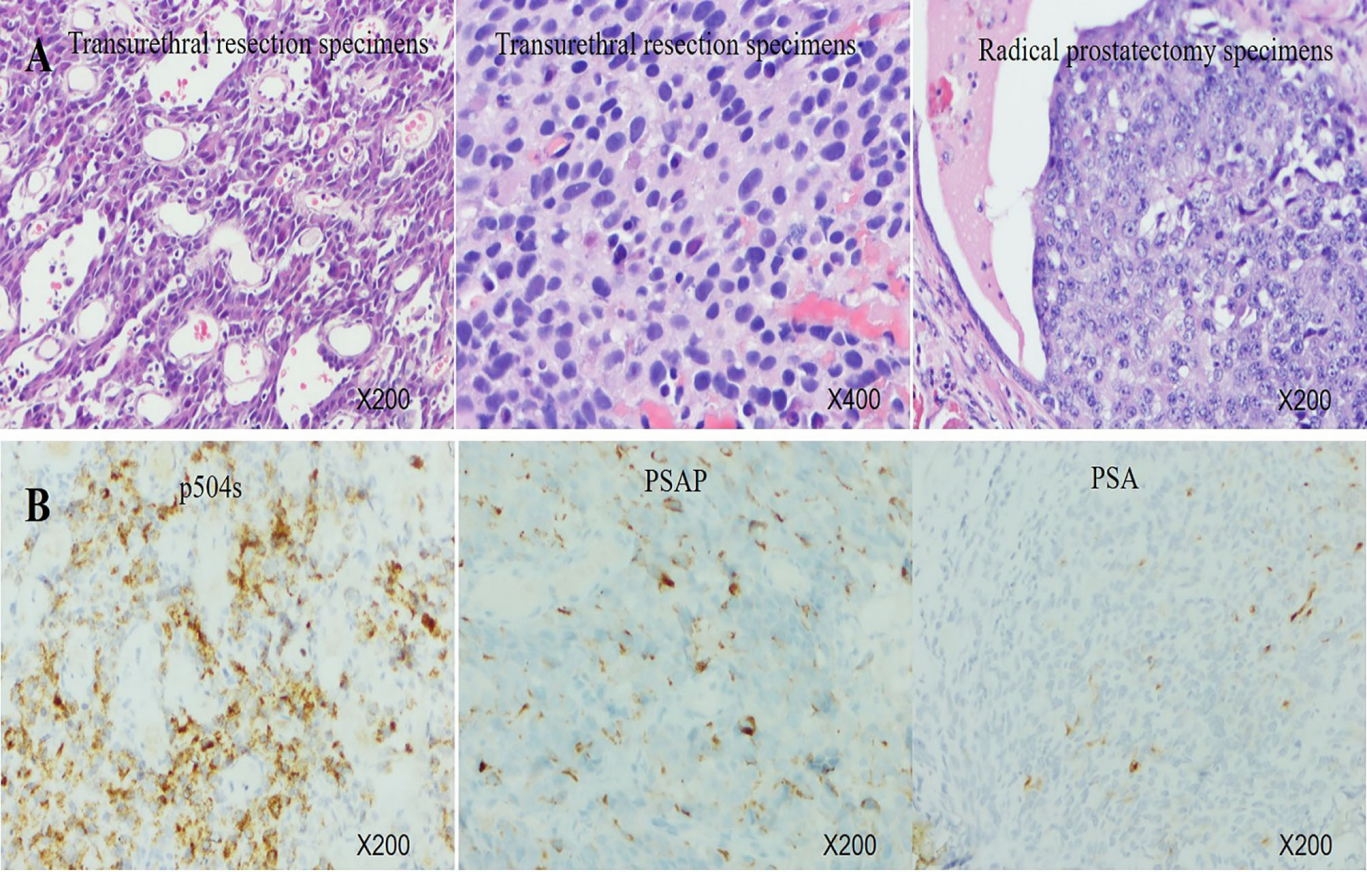
**(A)** Histology of transurethral resection specimens and radical prostatectomy specimens. **(B)** Immunohistochemistry of transurethral resection specimens showed very strong expression of P504S, middle expression of PSAP, and focal positivity for PSA.

**Figure 2:**
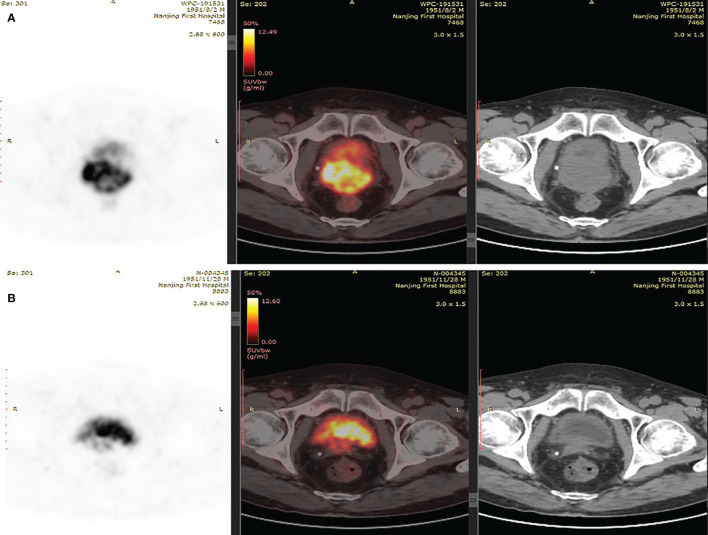
**(A)** PSMA-PET/CT scans suggested of uneven increase in PSMA expression of prostate. **(B)** PSMA-PET/CT scans showed shrunken lesions after platinum-based neoadjuvant chemotherapy.

A 67-year-old man was referred to the hospital due to frequency and urgency of urination, and difficulty of defecation. Cystoscopy reexamination showed no obvious abnormality in the bladder but irregular mass at the urethral orifice, indicating the possibility of PCa. The PSA level was 5.2 ng/ml. Transrectal ultrasound (TRUS) showed that the prostate was in irregular shape with an estimated volume of 84.6 ml. The boundary of the left lobe of the prostate with the rectum and the bilateral seminal vesicles was not clear. Pathological examination and immunohistochemistry of the tumor tissue from transurethral resection demonstrated that it was PCa adenocarcinoma with a Gleason score 10 (5 + 5) (**Figures 1A, B****)**. Results of prostate-specific membrane antigen (PSMA)-PET/CT scan showed a high level of asymmetrical *PSMA* uptake in the prostate, confirming invasion of the seminal vesicle and rectum (**Figure 2A**). The final clinical American Joint Committee on Cancer (8th edition) staging was IIIC (cT4, N0, cM0).

**Figure 3:**
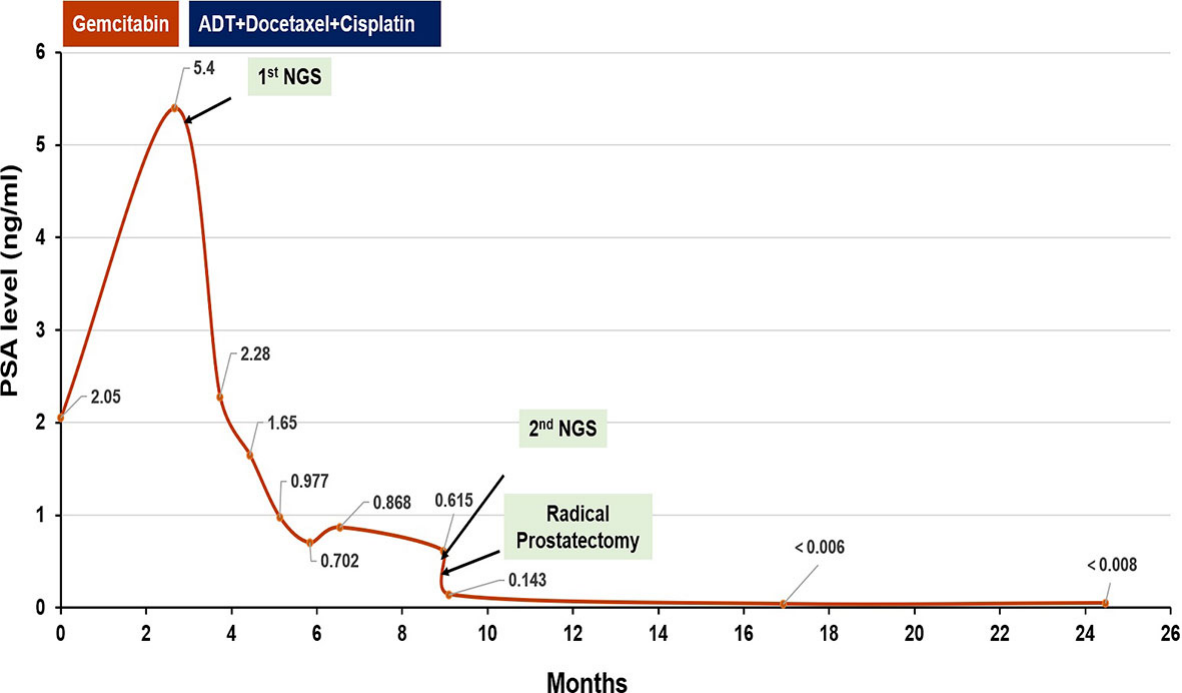
The clinical treatment course and prostate-specific antigen response.

As the patient had high Gleason Score and low PSA level, we performed next-generation sequencing (NGS) on his blood sample. The results revealed several pathogenic mutations in the HR pathway including BRCA2 p.F2234fs with a mutant allele frequency (MAF) of 1.2%, RAD51C c.905-2A>G (MAF, 1%). As the patient refused radiotherapy, neoadjuvant therapy combined with RP was considered. Due to NGS detection of several somatic pathogenic mutations in the HR pathway, he participated in a clinical trial (NCT04869371) and commenced on androgen deprivation therapy (ADT) plus platinum-based neoadjuvant chemotherapy (docetaxel 75 mg/m^2^ plus cisplatin 130 mg/m^2^) on October 11, 2019 (**Figure 3**). After six courses of treatment, his PSA level decreased to 0.615 ng/ml, and his symptoms of frequency and urgency of urination were relieved. The adverse reactions during neoadjuvant therapy mainly include fatigue, poor appetite, and diarrhea. In addition, TRUS indicated that the size of the prostate was smaller with estimated volume of 34.3 ml. Furthermore, PSMA-PET/CT scan found that the lesions obviously shrank (**Figure 2B**). Meanwhile, a second NGS was performed on the patient’s blood sample. Compared with the result of the first NGS, all the pathogenic mutations in the HR pathway were undetectable, and no new pathogenic mutations emerged.

On February 26, 2020, the patient underwent robotic-assisted RP and enlarged pelvic lymph node dissection. The surgical pathology revealed that it was prostate adenocarcinoma, with reduced tumor stage of IIIB (ypT3b, N0, cM0) (**Figure 1A**). The surgical margin and pelvic lymph node were negative. No adjuvant therapy was used after RP. At final follow-up (June 3, 2021), his PSA was still undetectable (<0.008 ng/ml) with normal level of testosterone, and no imaging progression was found by electroconvulsive therapy (ECT) and MRI scan (**Figure 3**).

## Discussion

High-risk PCa treated with curative intent is at increased risk of experiencing PSA failure, metastatic progression, and cancer-related death. The existence of micro-metastases disease is one of the main causes of this phenomenon. Neoadjuvant treatment not merely improves the function of traditional radical therapies but eliminates minimal residual disease to avoid tumor recurrence and metastatic progression. This has been proven by other solid cancers (13–15).

To improve surgical efficacy, neoadjuvant treatment strategies also have been employed in patients with high risk PCa. Shelley et al. performed meta-analysis of randomized trials of neoadjuvant hormone therapy (NHT) for localized or locally advanced PCa during 1966–2007. The results showed that NHT prior to RP significantly reduced positive margin rates, grade of tumor, and lymph node invasion but failed to improve overall disease-free survival (16). Nowadays, docetaxel or second-generation androgen receptor inhibitors combined with ADT have been investigated in neoadjuvant settings, as they result in significant improvement of overall survival in metastatic hormone-sensitive PCa (17–20). So far, several studies reported that neoadjuvant chemotherapy with docetaxel plus ADT was feasible and showed positive results for survival in high-risk PCa (21–23). However, the final outcomes for neoadjuvant chemotherapy with docetaxel have been inconclusive, which need more powerful evidence. Recently, neoadjuvant abiraterone or enzalutamide was reported to result in favorable pathological responses in some patients with high-risk disease (24, 25). Some pooled studies had indicated the better clinical oncological outcomes transferred from favorable pathological responses under neoadjuvant therapy of intense hormone therapy (26, 27). Although the efficacy of neoadjuvant therapies have not been determined in high-risk PCa patients, it is hopeful to witness final oncological benefits. In this regard, the identification of those patients who can benefit the most from such treatments is crucial.

PCa is a highly heterogeneous tumor even at its local stage. Neoadjuvant treatment of PCa is still focusing on intensive hormone therapy and thus lack of individualization, which may result in the limitation of complete pathological response (27). Precision and individualized treatment has been practiced in advanced PCa, but it can also be used for localized diseases. HRD is an important biomarker for precision therapy, which also leads to poor efficacy of traditional intensive hormone therapy (5, 6). Recent studies have reported that HRD are associated with platinum sensitivity in metastatic PCa. A retrospective study analyzed the association between germline BRCA2 variants and PSA response to carboplatin-based chemotherapy in a cohort of 141 men with metastatic castration-resistant PCa (mCRPC). Results suggested that pathogenic germline BRCA2 carriers had a higher response rate to carboplatin-based chemotherapy than non-BRCA2 carriers (10). Zafeiris Zafeiriou et al. presented three mCRPC cases with HRD who experienced impressive and durable responses to carboplatin (7). Another study presented that mCRPC patients with deleterious alterations in BRCA2, BRCA1, ATM, PALB2, FANCA, or CDK12 increased likelihood of achieving a PSA50 response and had longer time on platinum chemotherapy (8). More recently, Liancheng Fan et al. revealed that mCRPC harboring alterations in different genes of HR pathway displayed distinct response to platinum-based chemotherapy, and patients with BRCA2 and ATM alterations might experience more superior outcomes to platinum-based chemotherapy compared with other HR genes carriers (9).

In our case, the patient was with low PSA level and high Gleason Score at diagnosis. PSMA-PET/CT scan confirmed invasion of his seminal vesicle and rectum. Results of NGS analysis revealed pathogenic somatic mutations in the HR pathway. All of the above features suggested that the patient seemed to be more prone to micro-metastases and produce rapid clinical progression. Thus, a more aggressive treatment, ADT plus platinum-based neoadjuvant chemotherapy combined with RP, was finally chosen for him. After six cycles of treatment, the patient got better with decreased PSA level (0.615 ng/ml), reduced prostate size (from 84.6 to 34.3 ml), and diminished clinical symptoms. In addition, his second NGS results on blood sample collected after neoadjuvant therapy demonstrated that pathogenic mutations in the HR pathway disappeared, indicating tumor cell destroyed by platinum-based chemotherapy. Furthermore, 15 months after RP, the patient remains in clinical benefit and stable disease without increase in PSA and imaging progression. To our knowledge, this is the first case to report locally advanced PCa with HRD exhibiting outstanding response to platinum-based chemotherapy in neoadjuvant setting.

## Conclusion

We reported a patient with locally advanced PCa with HRD presenting pathological response and long duration of stable disease with no biochemical recurrence or imaging appearance of metastasis to platinum-based neoadjuvant chemotherapy. This work indicates a potential role for platinum-based neoadjuvant chemotherapy in the treatment of locally advanced PCa with HRD and advocate that treatment decisions can be individually tailored, possibly improving patient outcomes.

## Data Availability Statement

The original contributions presented in the study are included in the article/supplementary material. Further inquiries can be directed to the corresponding author.

## Ethics Statement

Ethical review and approval was not required for the study on human participants in accordance with the local legislation and institutional requirements. The patients/participants provided their written informed consent to participate in this study. Written informed consent was obtained from the individual(s) for the publication of any potentially identifiable images or data included in this article.

## Author Contributions

Conception and design of the work: JZ, SZ, XQ, YF, SA, TZ, YY, and HG. Supervision and writing of the paper: JZ. All authors contributed to the article and approved the submitted version.

## Conflict of Interest

TZ and YY are employed by GloriousMed Clinical Laboratory (Shanghai) Co., Ltd.

The remaining authors declare that the research was conducted in the absence of any commercial or financial relationships that could be construed as a potential conflict of interest.

## Publisher’s Note

All claims expressed in this article are solely those of the authors and do not necessarily represent those of their affiliated organizations, or those of the publisher, the editors and the reviewers. Any product that may be evaluated in this article, or claim that may be made by its manufacturer, is not guaranteed or endorsed by the publisher.

## Abbreviations

PCa, prostate cancer; PSA, prostate-specific antigen; RP, radical prostatectomy; HR, homologous recombination; HRD, homologous recombination defect; TRUS, transrectal ultrasound; NGS, next-generation sequencing; MAF, mutant allele frequency; ADT, androgen deprivation therapy; NHT, neoadjuvant hormone therapy; OS, overall survival; mCRPC, metastatic castration-resistant prostate cancer.
